# Supplementary material for: Ultra-low friction graphene oxide in the Atotsugawa Fault System
Source: Nat Commun. 2026 May 12;17:3861. doi: 10.1038/s41467-026-72239-5 (PMC13168515; doi:10.1038/s41467-026-72239-5)
Supplement: Supplementary file 1 — Supplementary Information [file 41467_2026_72239_MOESM1_ESM.pdf]

Supplementary Information
Contents of this file

Fig. S1. Detailed topographic maps showing the locations of sampling sites.
Fig. S2. Outcrop sketch in the Active Fault Survey Tunnel (AFST), which penetrates the B fracture zone of the Mozumi–Sukenobe Fault (modified from ref. 43).
Fig. S3. Outcrop photographs of samples 1535 and 1540 in the Sengoku area.
Fig. S4. Outcrop photograph of sample 1323 in the Kiritani area.
Fig. S5. Outcrop photographs of samples 1424, 1435, 1443 and 1506 in the Inotani area.
Fig. S6. Outcrop photographs of samples 1105 and 1236 in the Sukenobe area.
Fig. S7. Outcrop photograph in the AFST, which penetrates the B fracture zone of the Mozumi–Sukenobe Fault.
Fig. S8. Outcrop photographs of samples 1124 and 1141 in the Atotsugawa area.
Fig. S9. X-ray diffraction (XRD) pattern of the AFST fault gouge sample.
Fig. S10. Line analysis by X-ray photoelectron spectroscopy (XPS) across fault gouge and microcracks from region 9 of sample AFST.
Fig. S11. TEM image (yellow dashed region in Fig. 6a) and corresponding EDS element maps overlain on the TEM image.
Fig. S12. SAED pattern images collected from location 9 and 19 in Fig. 6a.
Table S1. Summary of lithologies in the Tetori Group around the Atotsugawa Fault System.
Table S2. Peaks in the Raman spectral bands of rocks from the Sengoku area.
Table S3. Peaks in the Raman spectral bands of rocks from the Inotani area.
Table S4. Peaks in the Raman spectral bands of rocks from the Sukenobe area.
Table S5. Peaks in the Raman spectral bands of rocks from the Active Fault Survey Tunnel (sample AFST).
Table S6. Peaks in the Raman spectral bands of rocks from the Atotsugawa area.
Reference

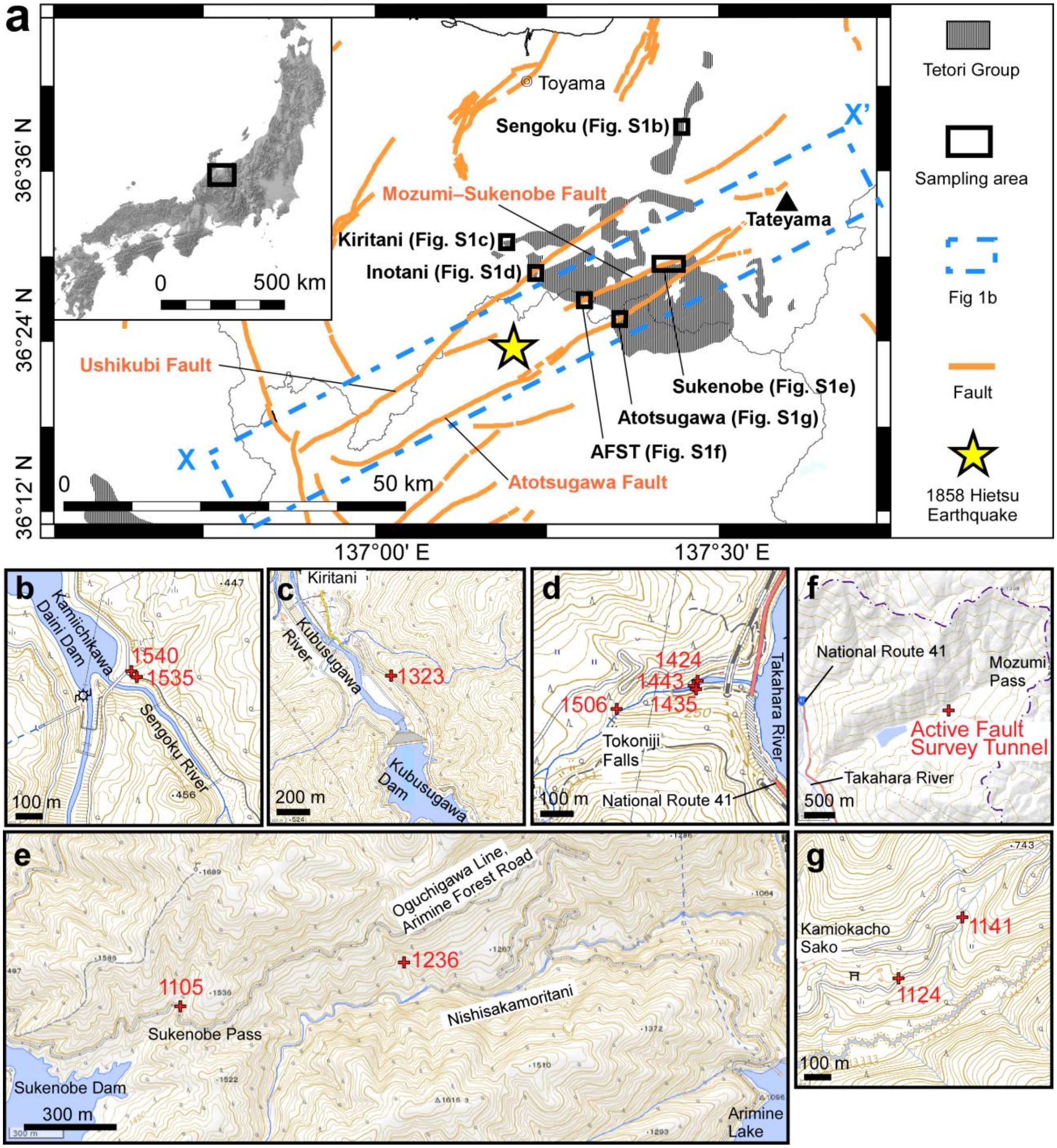

Fig. S1. Detailed topographic maps of the locations of sampling sites. a, Summary geological map of the Atotsugawa Fault System, modified after the Visualization System for Subsurface Structures (https://gbank.gsj.jp/activefault/index_e_gmap.html)[70]. Hatched areas = exposures of the Tetori Group; orange bold lines = faults; black rectangles = study areas; yellow star = hypocenter of the 1858 Hietsu earthquake[6, 7]; AFST = Active Fault Survey Tunnel through the Mozumi-Sukenobe Fault. **b–g,** topographic maps from the Geospatial Information Authority of Japan showing the locations of sampling sites (modified from ref. 71, https://maps.gsi.go.jp/). **b,** Sengoku area. **c,** Kiritani area. **d,** Inotani area. **e,** Sukenobe area. **f,** Active Fault Survey Tunnel (AFST). **g,** Atotsugawa area. Table S1 lists the locations of the sampling sites.

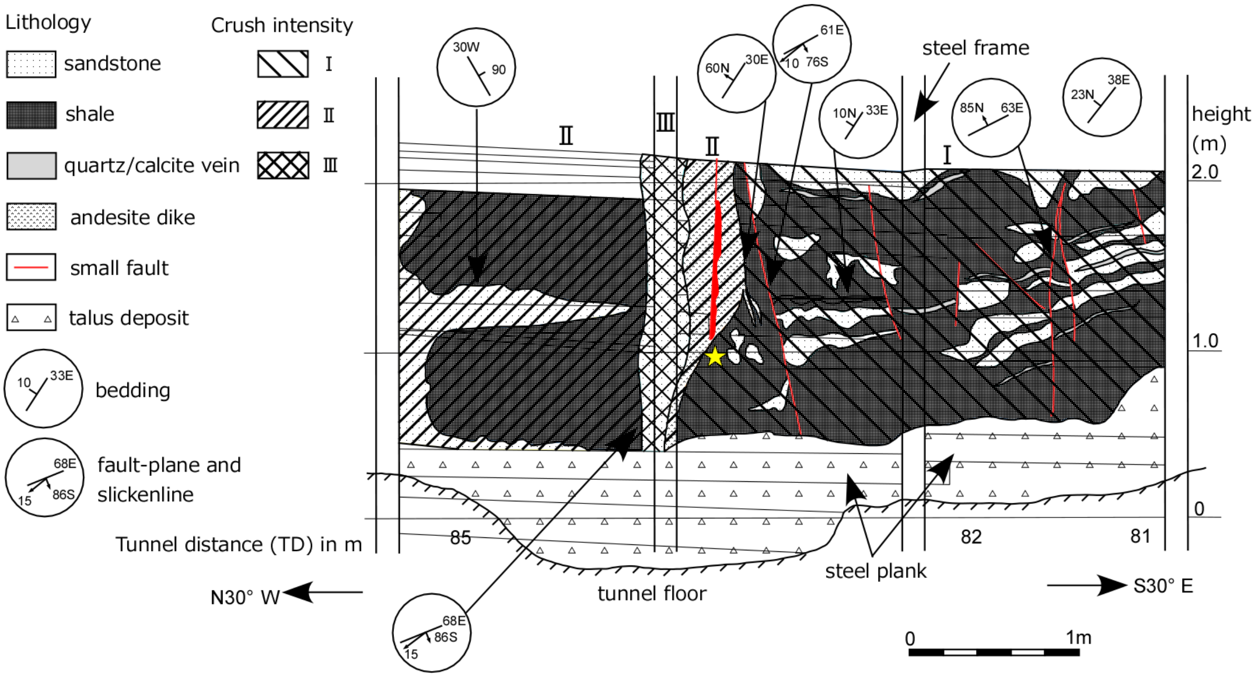

Fig. S2. Outcrop sketch in the Active Fault Survey Tunnel (AFST), which penetrates the B fracture zone of the Mozumi–Sukenobe Fault (modified from ref. 43). The sample (yellow star) of fault gouge was collected from the B fracture zone on the southern side of the AFST (Fig. S1f). The sampling site is located in shales of the Tetori Group (Upper Jurassic to Lower Cretaceous sedimentary rocks) and the sample was collected approximately 20 cm from the most intensely fractured zone (zone III; N68°E, 86°S)[37, 43].

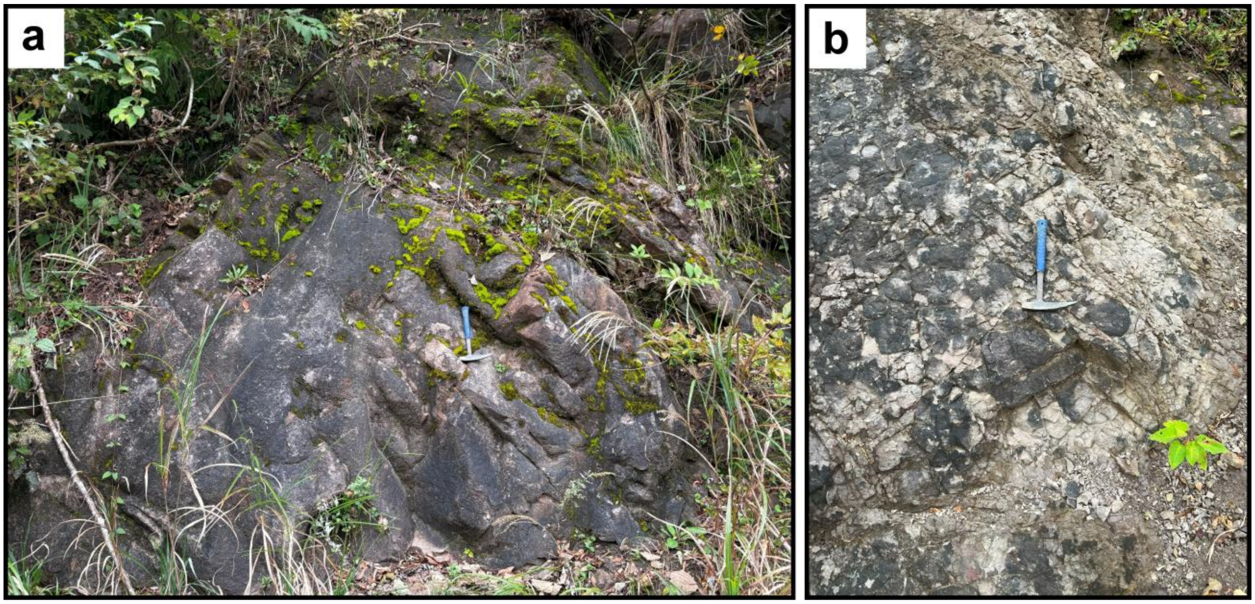

Fig. S3. Outcrop photographs of samples 1535 and 1540 in the Sengoku area. a, sample 1535. **b,** sample 1540. A hammer (approximately 30 cm long) is included for scale. The outcrop is along a forest road upstream of the Kamiichikawa Daini Dam on the Sengoku River (near Kamiichi Town, Toyama Prefecture; Fig. 1a and Fig. S1b). Sample 1535 is a very coarse-grained sandstone (Table S1). Sample 1540 is located a few meters closer to the dam and is a coarse-grained sandstone (Table S1).

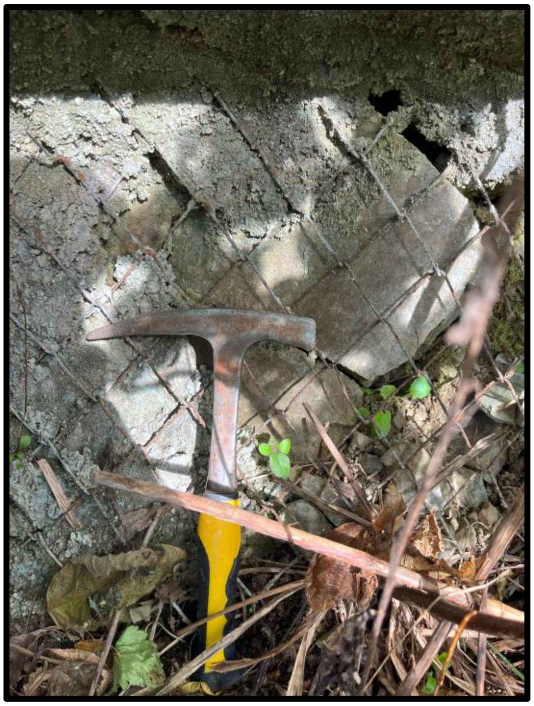

Fig. S4. Outcrop photograph of sample 1323 in the Kiritani area. The outcrop is located on an eastward-facing slope along the road north of the Kubusugawa Dam (Kubusugawa River, Toyama City; Fig. S1c). Sample 1323 is a medium-grained sandstone (Table S1).

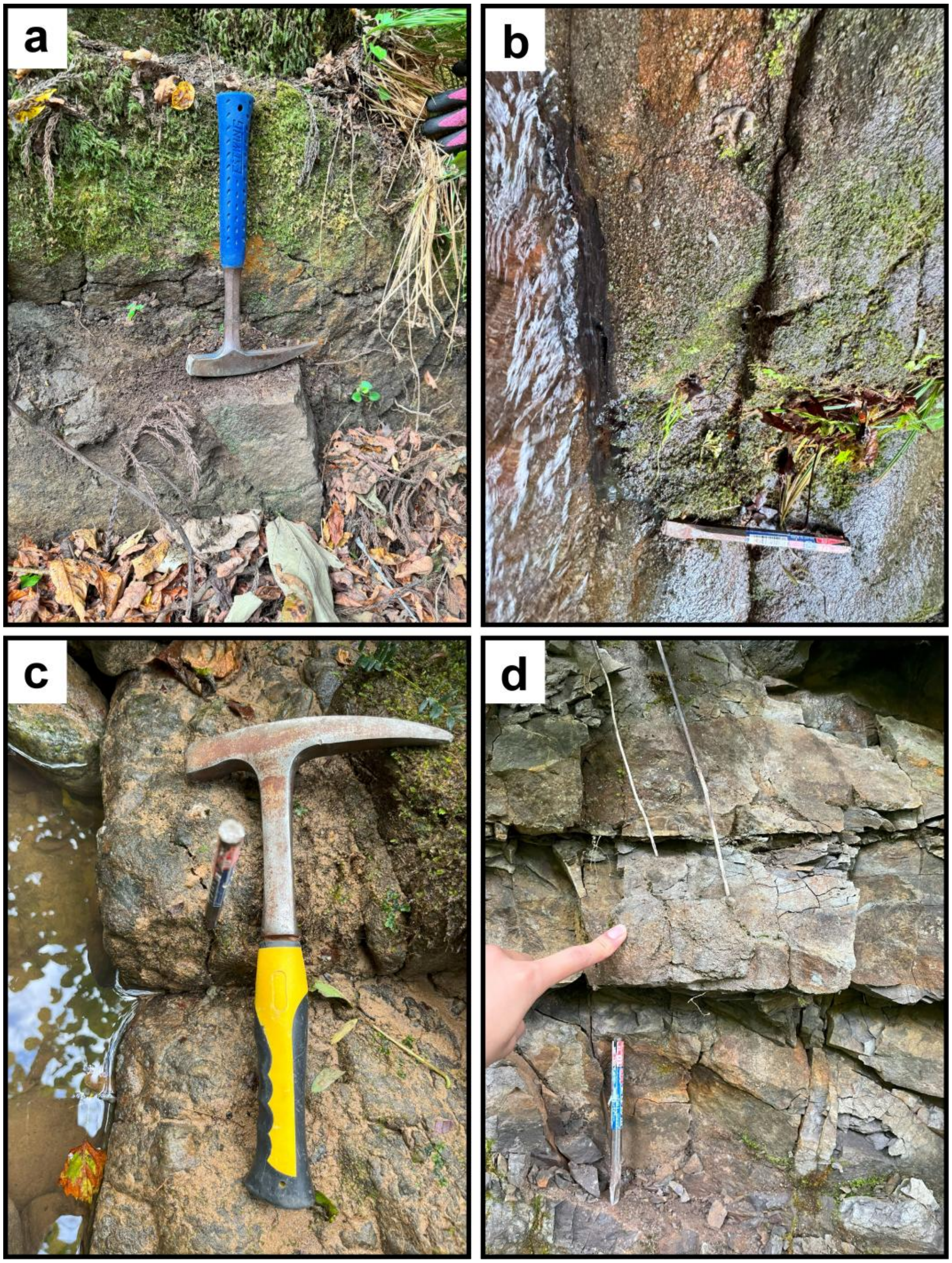

Fig. S5. Outcrop photographs of samples 1424, 1435, 1443 and 1506 in the Inotani area. a, sample 1424. **b,** sample 1435. **c,** sample 1443. **d,** sample 1506. A hammer (approximately 30 cm long) and chisel (approximately 10 cm long) are included for scale. The outcrops are located near Tokoniji Falls in Inotani, Toyama City, on the western side of National Route 41 (Fig. S1d). The samples belong to the lower part of the Inotani alternation member[38, 42]. Samples 1424, 1435, and 1443 were collected from an outcrop approximately 260 m east of Tokoniji Falls. Sample 1424 is a granular conglomerate that was collected on a nearby mountain slope (Table S1). Sample 1435 was collected from a river bed and comprises a granular conglomerate with clasts up to ~10 mm in size (Table S1). Sample 1443 was collected north of sample 1435 and comprises a pebble conglomerate (Table S1). Sample 1506 was collected near Tokoniji Falls and comprises a very coarse-grained sandstone with bedding planes oriented N80°E, 4°N (Table S1).

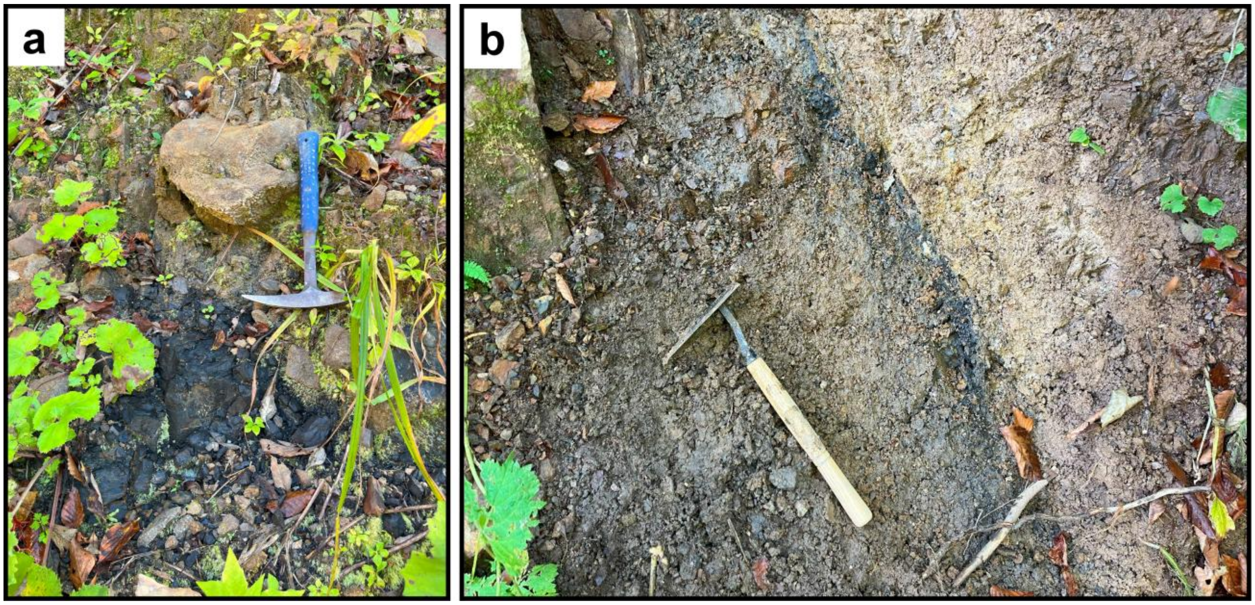

Fig. S6. Outcrop photographs of samples 1105 and 1236 in the Sukenobe area. a, sample 1105. **b,** sample 1236. A hammer (approximately 30 cm long) and sickle (approximately 25 cm long) are included for scale. The outcrops are located on the eastern side of the Mozumi-Sukenobe Fault, near Lake Arimine and Sukenobe Dam in Toyama City (Fig. 1a and Fig. S1e). Sample 1105 was collected from an outcrop along a forest road about 170 m northwest of Sukenobe Pass. The sample contains very coarse-grained sandstone and black mudstone (Fig. S1e and Table S1). The lithologies are subvertically inclined and weakly crushed due to intermittent post-Cretaceous faulting[8]. Sample 1236 was collected from a fault at Nishisakamoritani (Ashitani), about 500 m west of a hairpin bend on the Oguchigawa Line of the Arimine Forest Road. The lithologies in sample 1236 comprise dark gray and black fault gouge containing clay-sized particles, sandwiched by coarse-grained sandstone (Table S1). The fault gouge does not exhibit notable heterogeneity in color or composition.

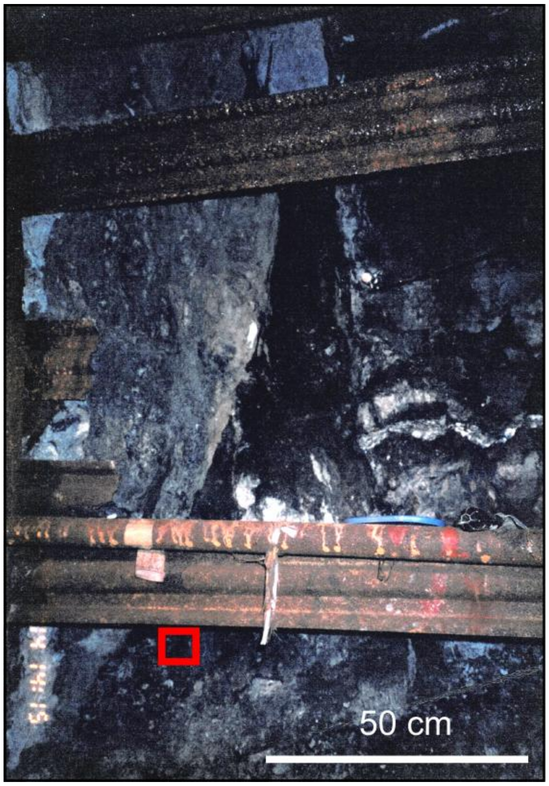

Fig. S7. Outcrop photograph in the AFST, which penetrates the B fracture zone of the Mozumi–Sukenobe Fault. The red box shows the location of the fault gouge sample (Table S1).

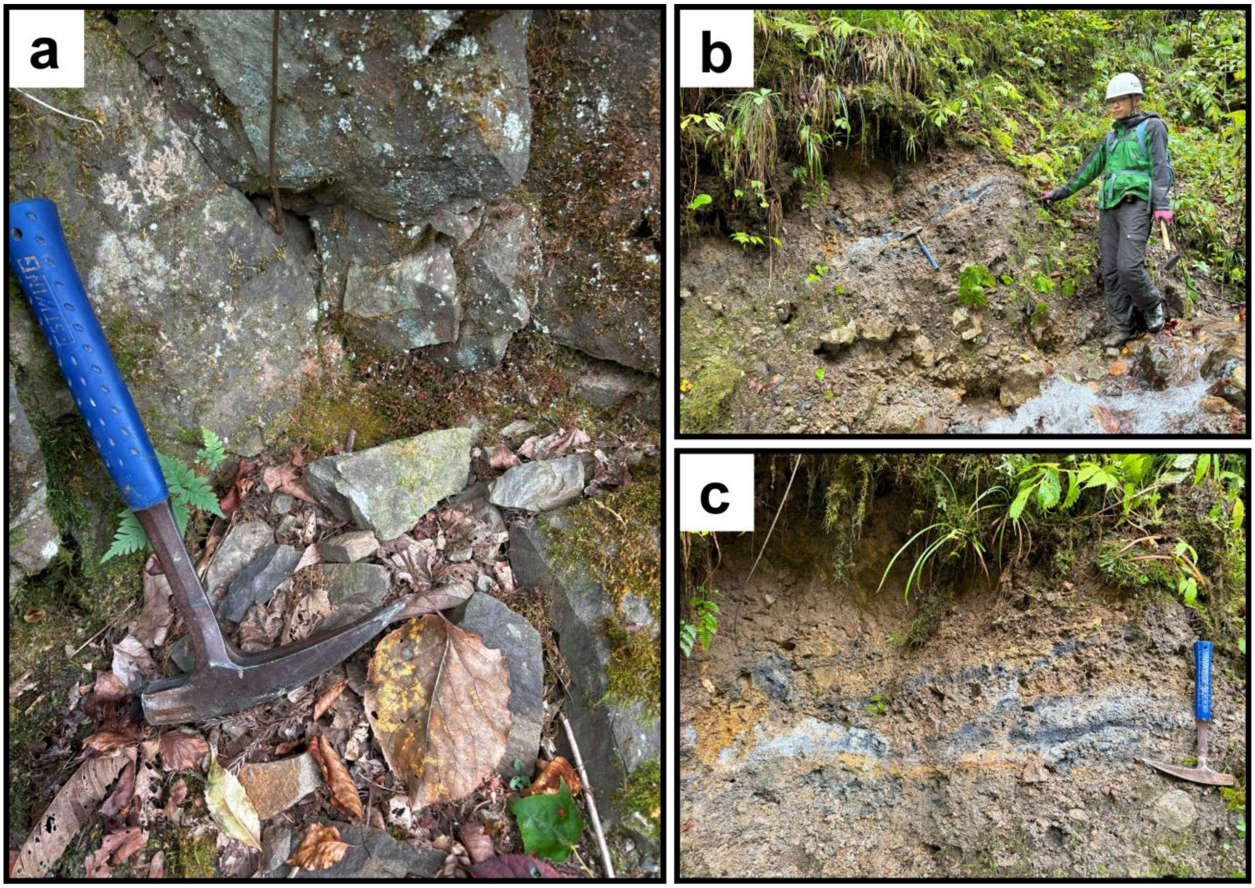

Fig. S8. Outcrop photographs of samples 1124 and 1141 in the Atotsugawa area. a, sample 1124. **b–c,** sample 1141. A hammer (approximately 30 cm long) is included for scale. The outcrops are located in Kamiokacho Sako, Hida City, Gifu Prefecture (Fig. 1a and Fig. S1g). Sample 1124 was collected from an outcrop along a forest road near a shrine in Sako, and comprises a medium-grained sandstone (Table S1). Sample 1141 was collected in a valley near a hairpin bend in the forest road, approximately 400 m northeast of the shrine (Fig. S1g). Sample 1141 is a clay-rich, black and gray fault gouge from an outcrop of the Atotsugawa Fault (Table S1). The fault gouge displays notable black and gray color banding, with the black gouge being finer-grained than the gray gouge.

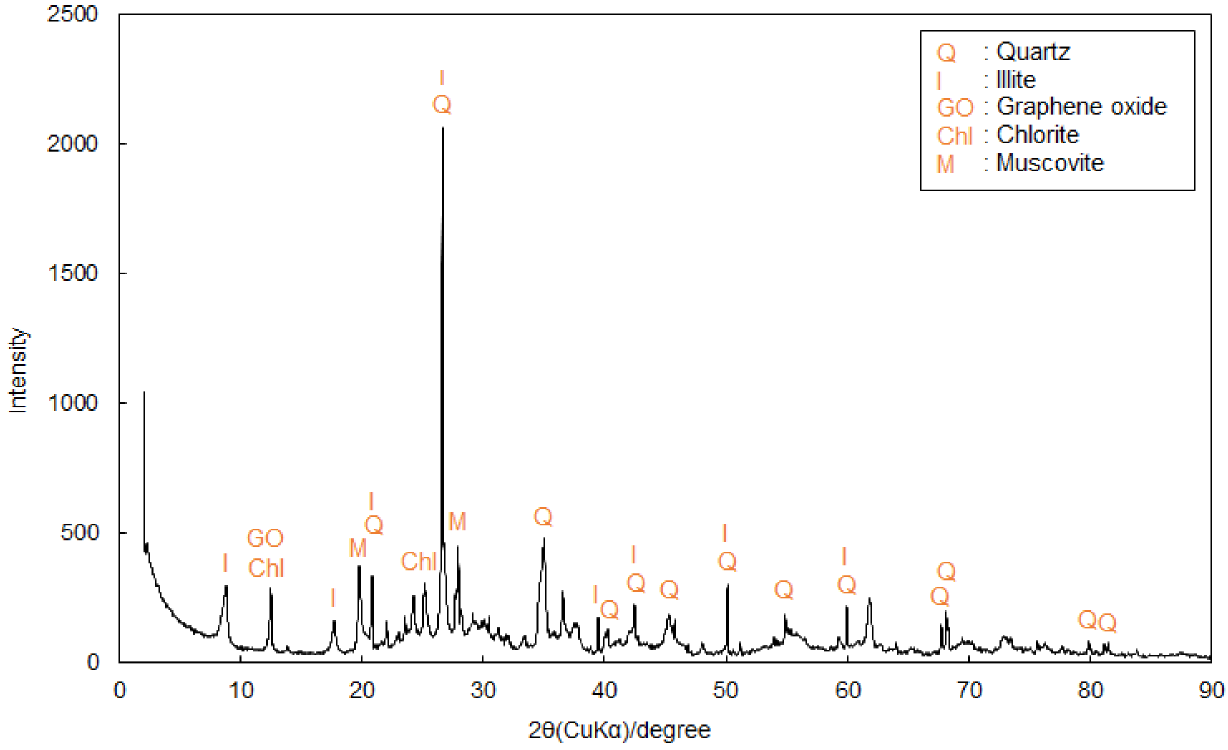

Fig. S9. X-ray diffraction (XRD) pattern of the AFST fault gouge sample. Q = quartz; I = illite; GO = graphene oxide; Chl = chlorite; M = muscovite. X-ray diffraction (XRD) analysis was performed using an automated multipurpose X-ray diffractometer (Philips, X'Pert-PRO) in the Department of Earth Science, Tohoku University, Sendai, Japan. Measurements were conducted on powdered fault gouge sample under conditions of 40 kV and 50 mA. Mineral identification based on XRD peaks was carried out with reference to TEM observations and ref. 39.

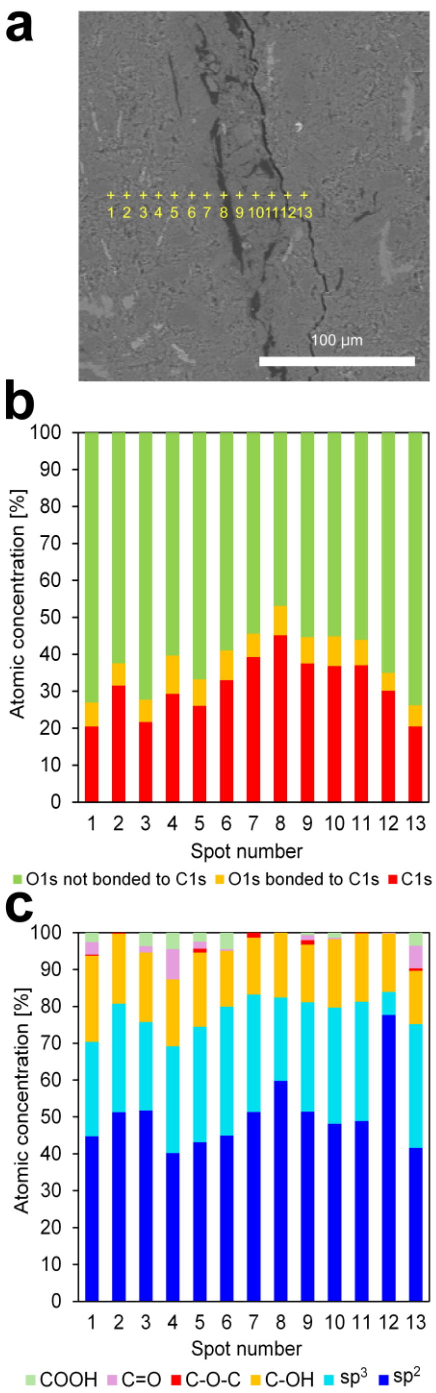

Fig. S10. Line analysis by X-ray photoelectron spectroscopy (XPS) across fault gouge and microcracks from region 9 of sample AFST. See Fig. 2f for location. **a,** Scanning X-ray image of region 9. **b,** Atomic concentration of carbon and oxygen in the measured points. **c,** Concentration of chemical bonding in the measured points. Line analysis was performed across regions of intact gouge associated with networks of microcracks (Fig. S10a). Fig. S10b shows the concentration of carbon and oxygen at each measurement point. The C 1*s* peaks show a distinctly increased concentration within the microcracks (Fig. S10b). Fig. S10c shows the concentration of carbon bonds and oxygen-containing functional groups. At spots 8 and 12, microcracks ~5 µm wide contain a relatively high proportion of sp^2 bonds and epoxy groups are absent (Fig. S10c). The carbon in these microcracks is identified as CM characterized by sp^2 structures bonded with oxygen-containing functional groups, without any contribution from epoxy resin.

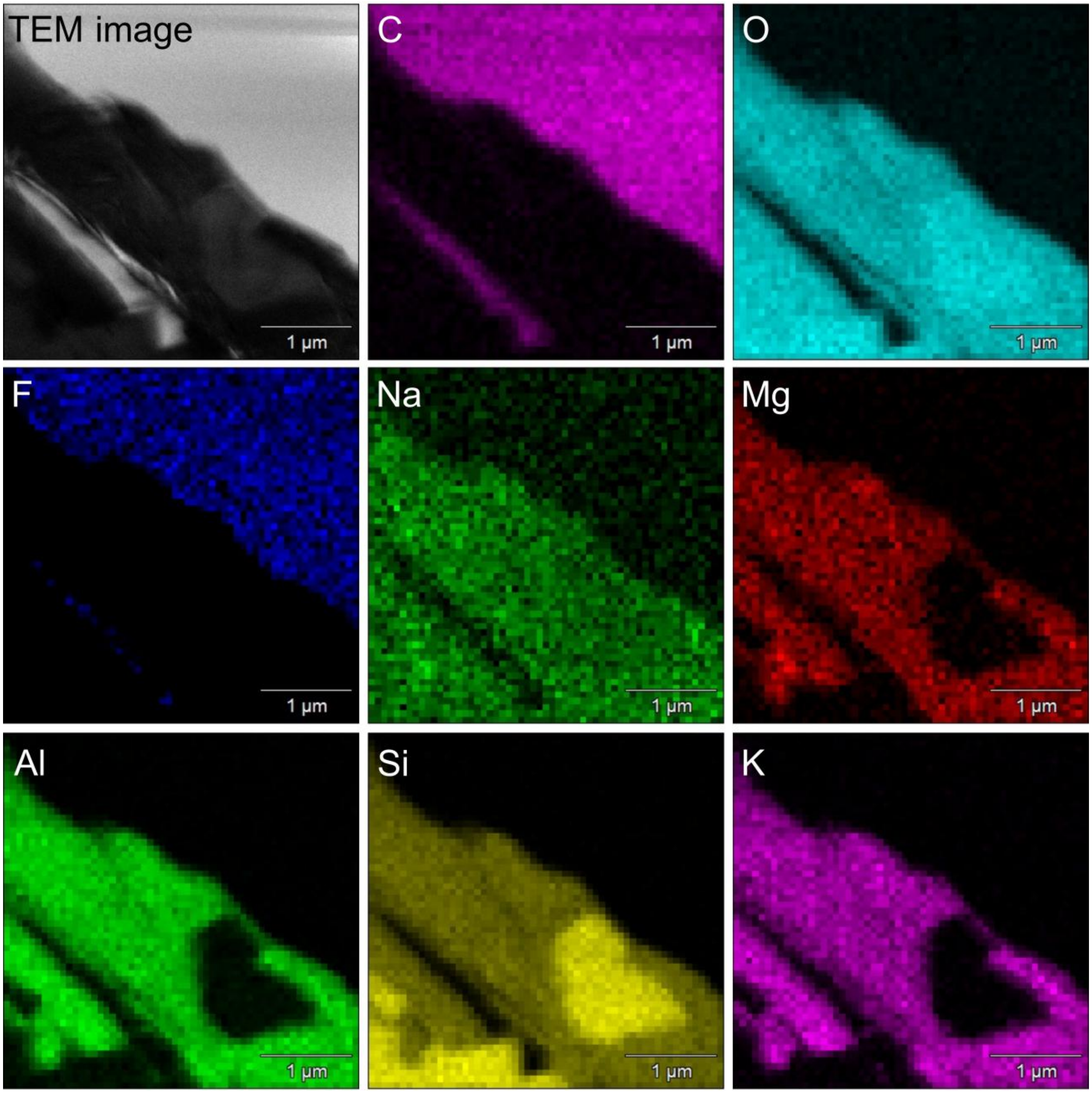

Fig. S11. TEM image (yellow dashed region in Fig. 6a) and corresponding EDS element maps overlain on the TEM image. The carbon abundance map reveals regions where graphene oxide is concentrated. The oxygen abundance map represents relative amounts, which indicates that both carbon and oxygen are abundant (also see the EDS spectrum in Fig. 6b). In the silicon abundance map, the brightest regions are identified as quartz (by considering the composition of the other maps), while the regions with less silicon are identified as illite.

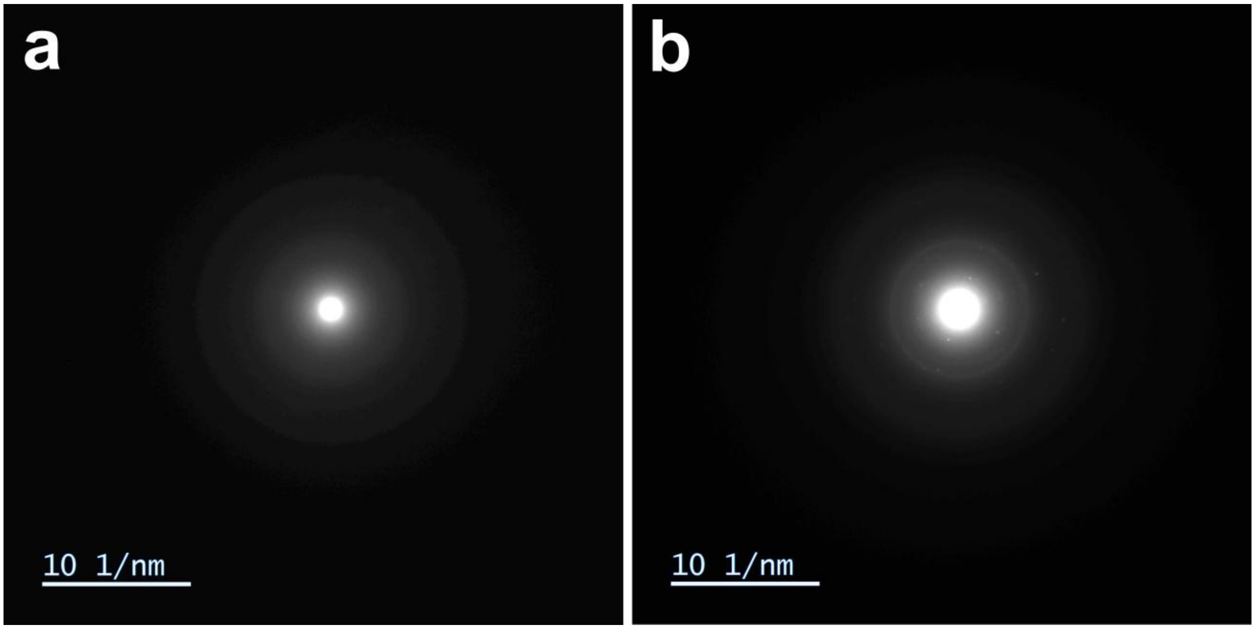

Fig. S12. SAED pattern images collected from location 9 and 19 in Fig. 6a. a, SAED pattern from location 9 contains a faint ring with a d-spacing of 0.21 nm, corresponding to the 100 reflection of graphene. **b,** SAED pattern collected from location 19 shows diffraction patterns attributable to both graphene and clay minerals. The interface between illite and CM has roughness (shown by yellow dashed region in Fig. 6a and Fig. S11). Therefore, these observations implies that graphene oxide and illite may be locally mixed. We suggest that some graphene oxide nanosheets can be arranged along the irregular interfaces with clay minerals, potentially resulting in variable orientations.

Table S1. Summary of lithologies in the Tetori Group around the Atotsugawa Fault System.

Area	Sample code	Coordinates of site	Lithologies	Other comments
Sengoku	1535	36° 39′ 25.01″ N, 137° 26′ 47.39″ E	Host rock, very coarse sandstone	-
Sengoku	1540	36° 39′ 25.51″ N, 137° 26′ 46.51″ E	Host rock, coarse sandstone	-
Kiritani	1323	36° 30′ 50.08″ N, 137° 11′ 10.95″ E	Host rock, medium sandstone	-
Inotani	1424	36° 28′ 41.34″ N, 137° 13′ 54.36″ E	Host rock, granule conglomerate	-
Inotani	1435	36° 28′ 40.47″ N, 137° 13′ 53.94″ E	Host rock, granule conglomerate	-
Inotani	1443	36° 28′ 40.76″ N, 137° 13′ 54.27″ E	Host rock, pebble conglomerate	-
Inotani	1506	36° 28′ 38.22″ N, 137° 13′ 44.61″ E	Host rock, very coarse sandstone	-
Sukenobe	1105a	36° 29′ 27.09″ N, 137° 24′ 37.67″ E	Host rock, very coarse sandstone	-
Sukenobe	1105b	36° 29′ 27.09″ N, 137° 24′ 37.67″ E	Host rock, black mudstone	-
Sukenobe	1236a	36° 29′ 33.29″ N, 137° 25′ 27.18″ E	Host rock, coarse sandstone	North side
Sukenobe	1236b	36° 29′ 33.29″ N, 137° 25′ 27.18″ E	Host rock, coarse sandstone	South side
Sukenobe	1236e	36° 29′ 33.29″ N, 137° 25′ 27.18″ E	Fault gouge	Central part
Sukenobe	1236	36° 29′ 33.29″ N, 137° 25′ 27.18″ E	Fault gouge	Lower-left part
AFST	AFST	36° 26′ 41.40″ N, 137° 17′ 57.92″ E	Fault gouge	-
Atotsugawa	1124	36° 25′ 14.98″ N, 137° 20′ 45.71″ E	Host rock, medium sandstone	-
Atotsugawa	1141	36° 25′ 22.33″ N, 137° 20′ 55.46″ E	Fault gouge	

Table S2. Peaks in the Raman spectral bands of rocks from the Sengoku area.

Sample	Spot	D [cm^{-1}]	G_{app} [cm^{-1}]	2D [cm^{-1}]	D + D' [cm^{-1}]	2D' [cm^{-1}]	D'_{inf} [cm^{-1}]	D'_{inf} - G_{app} [cm^{-1}]	Species
1535	1	1325.5	1629.2	-	-	-	-	-	AC[c]
	2	-	-	-	-	-	-	-	Non carbon
	3	-	-	-	-	-	-	-	Non carbon
	4	1327.3	1596.3	-	-	-	-	-	AC[c]
	5	-	-	-	-	-	-	-	Non carbon
	6	1363.8	1603.1	-	-	-	-	-	AC[c]
	7	1469.1	1605.2	-	-	-	-	-	AC[c]
	8	1417.7	1599.3	-	-	-	-	-	AC[c]
	9	1357.3	1599.7	-	-	-	-	-	AC[c]
	10	1289.9	1597.6	-	-	-	-	-	AC[c]
1540	1	-	-	-	-	-	-	-	Non carbon
	2	-	-	-	-	-	-	-	Non carbon
	3	-	-	-	-	-	-	-	Non carbon
	4	-	-	-	-	-	-	-	Non carbon
	5	-	-	-	-	-	-	-	Non carbon
	6	1377.6	1598.6	-	-	-	-	-	AC[c]
	7	-	-	-	-	-	-	-	Non carbon
	8	1340.8	1601.0	-	-	-	-	-	AC[c]
	9	-	-	-	-	-	-	-	Non carbon
	10	-	-	-	-	-	-	-	Non carbon

AC[c], Amorphous carbon without an identifiable 2D′ peak

Table S3. Peaks in the Raman spectral bands of rocks from the Inotani area.

Sample	Spot	D [cm^{-1}]	G_{app} [cm^{-1}]	2D [cm^{-1}]	D + D' [cm^{-1}]	2D' [cm^{-1}]	D'_{inf} [cm^{-1}]	D'_{inf} - G_{app} [cm^{-1}]	Species
1424	1	1364.7	1604.0	2650.8	2919.3	3161.7	1580.9	-23.2	GOCM[a]
	2	1325.4	1603.5	2656.2	2946.1	3174.6	1587.3	-16.2	GOCM[a]
	3	1344.4	1598.3	-	-	-	-	-	AC[c]
	4	-	-	-	-	-	-	-	Non carbon
	5	1342.6	1603.1	2691.6	2986.2	3238.4	1619.2	16.1	RGOCM[b]
	6	-	-	-	-	-	-	-	Non carbon
	7	-	-	-	-	-	-	-	Non carbon
	8	1365.2	1602.1	-	2870.0	-	-	-	AC[c]
	9	-	-	-	-	-	-	-	Non carbon
	10	-	-	-	-	-	-	-	Non carbon
1435	1	1352.8	1600.4	2649.6	2927.1	3213.1	1606.6	6.1	RGOCM[b]
	2	-	-	-	-	-	-	-	Non carbon
	3	1348.6	1600.2	-	3018.4	-	-	-	AC[c]
	4	1349.4	1602.4	2730.1	2968.5	3243.8	1621.9	19.5	RGOCM[b]
	5	1380.9	1601.5	-	-	-	-	-	AC[c]
	6	1444.1	1602.1	2702.4	2973.9	3244.6	1622.3	20.2	RGOCM[b]
	7	-	-	-	-	-	-	-	Non carbon
	8	1350.0	1602.0	2584.8	2910.9	3204.4	1602.2	0.2	RGOCM[b]
	9	1330.1	1600.5	2683.0	2883.5	3216.5	1608.2	7.7	RGOCM[b]
	10	-	-	-	-	-	-	-	Non carbon
1443	1	1338.6	1601.6	2651.4	2945.6	3212.9	1606.4	4.8	RGOCM[b]
	2	1341.0	1603.2	2624.5	2906.4	3213.3	1606.7	3.5	RGOCM[b]
	3	-	-	-	-	-	-	-	Non carbon
	4	1337.9	1600.4	2615.9	2894.0	3224.5	1612.2	11.9	RGOCM[b]
	5	1336.9	1604.6	2644.3	3038.2	-	-	-	AC[c]
	6	1321.3	1606.8	-	-	-	-	-	AC[c]
	7	1338.5	1601.8	2610.6	2919.1	3219.2	1609.6	7.8	RGOCM[b]
	8	-	-	-	-	-	-	-	Non carbon
	9	-	-	-	-	-	-	-	Non carbon
	10	-	-	-	-	-	-	-	Non carbon
1506	1	1347.8	1604.3	2695.8	2943.2	3195.9	1598.0	-6.3	GOCM[a]
	2	-	-	-	-	-	-	-	Non carbon

3	1340.8	1604.4	-	2849.0	3189.1	1594.6	-9.9	GOCM[a]
4	-	-	-	-	-	-	-	Non carbon
5	-	-	-	-	-	-	-	Non carbon
6	1334.7	1603.1	2583.6	2888.1	3205.9	1603.0	-0.1	GOCM[a]
7	-	-	-	-	-	-	-	Non carbon
8	1334.4	1602.7	2645.4	2929.4	3202.8	1601.4	-1.3	GOCM[a]
9	1336.3	1602.9	2654.0	2912.7	3206.6	1603.3	0.4	RGOCM[b]
10	1332.7	1601.5	2655.2	2958.2	3215.2	1607.6	6.1	RGOCM[b]

GOCM[a], graphene oxide–like carbonaceous material
RGOCM[b], reduced graphene oxide–like carbonaceous material
AC[c], Amorphous carbon without an identifiable 2D′ peak

Table S4. Peaks in the Raman spectral bands of rocks from the Sukenobe area.

Sample	Spot	D [cm^{-1}]	G_{app} [cm^{-1}]	2D [cm^{-1}]	D + D' [cm^{-1}]	2D' [cm^{-1}]	D'_{inf} [cm^{-1}]	D'_{inf} - G_{app} [cm^{-1}]	Species
1105a	1	-	-	-	-	-	-	-	Non carbon
	2	1338.4	1601.1	-	-	-	-	-	AC[c]
	3	-	-	-	-	-	-	-	Non carbon
	4	-	-	-	-	-	-	-	Non carbon
	5	-	-	-	-	-	-	-	Non carbon
	6	1301.8	1598.7	-	-	-	-	-	AC[c]
	7	-	-	-	-	-	-	-	Non carbon
	8	-	-	-	-	-	-	-	Non carbon
	9	1328.9	1602.0	-	-	-	-	-	AC[c]
	10	1373.9	1594.3	-	-	-	-	-	AC[c]
1105b	1	1338.8	1602.2	-	-	-	-	-	AC[c]
	2	1340.5	1603.0	-	-	3198.0	1599.0	-4.0	GOCM[a]
	3	1353.4	1602.5	2585.0	2908.9	-	-	-	AC[c]
	4	1352.8	1603.5	-	-	-	-	-	AC[c]
	5	1339.6	1605.6	-	-	-	-	-	AC[c]
	6	1311.3	1604.7	-	-	3226.0	1613.0	8.3	RGOCM[b]
	7	1344.0	1602.8	2572.9	2857.3	3209.5	1604.8	2.0	RGOCM[b]
	8	1335.1	1603.8	2683.5	2964.7	3213.5	1606.7	2.9	RGOCM[b]
	9	1345.2	1603.3	2716.5	2942.4	-	-	-	AC[c]
	10	1340.6	1605.8	2786.8	-	-	-	-	AC[c]
1236a	1	1341.1	1603.7	2768.6	2954.3	3173.5	1586.8	-16.9	GOCM[a]
	2	1343.1	1602.3	-	2960.5	-	-	-	AC[c]
	3	1360.9	1602.2	-	2959.1	-	-	-	AC[c]
	4	-	-	-	-	-	-	-	Non carbon
	5	1340.7	1603.5	2687.8	2885.3	3197.2	1598.6	-4.9	GOCM[a]
	6	-	-	-	-	-	-	-	Non carbon
	7	1338.7	1603.7	2760.3	2942.9	3182.2	1591.1	-12.6	GOCM[a]
	8	1346.3	1601.9	2717.1	2947.8	3188.9	1594.5	-7.4	GOCM[a]
	9	1350.6	1603.2	2806.6	2953.3	3184.1	1592.0	-11.2	GOCM[a]
	10	1342.0	1603.6	2667.7	2911.2	3210.5	1605.2	1.6	RGOCM[b]
1236b	1	1341.6	1603.6	2667.8	2911.2	3210.5	1605.2	1.6	RGOCM[b]
	2	1339.2	1602.6	2647.2	2915.7	3180.9	1590.5	-12.1	GOCM[a]

	3	1367.3	1601.8	-	-	-	-	-	AC[c]
	4	-	-	-	-	-	-	-	Non carbon
	5	-	-	-	-	-	-	-	Non carbon
	6	-	-	-	-	-	-	-	Non carbon
	7	1320.4	1603.1	2646.0	2912.5	3180.0	1590.0	-13.1	GOCM[a]
	8	-	-	-	-	-	-	-	Non carbon
	9	-	-	-	-	-	-	-	Non carbon
	10	-	-	-	-	-	-	-	Non carbon
1236e	6	1313.7	1599.2	2632.8		3209.1	1604.5	5.3	RGOCM[b]
	7	1339.1	1604.3	2418.1	2705.4	3227.7	1613.8	9.6	RGOCM[b]
	9	1348.1	1605.9	2714.2	2966.8	3202.4	1601.2	-4.7	GOCM[a]
	10	1329.7	1603.9	2568.7	2809.7	3200.9	1600.5	-3.4	GOCM[a]
1236	1	1371.8	1593.4	2381.9	2867.3	3113.6	1556.8	-36.6	GOCM[a]
	2	1339.6	1603.4	2727.2	2958.1	3205.1	1602.6	-0.8	GOCM[a]
	3	1376.6	1593.4	2402.1	2899.7	3163.2	1581.6	-11.8	GOCM[a]
	4	1345.7	1600.8	2340.8	2893.0	3140.6	1570.3	-30.6	GOCM[a]
	5	1332.8	1603.4	2516.6	2937.0	3180.9	1590.4	-13.0	GOCM[a]
	6	1387.2	1597.9	2445.3	2916.4	3150.4	1575.2	-22.7	GOCM[a]

GOCM[a], graphene oxide–like carbonaceous material
RGOCM[b], reduced graphene oxide–like carbonaceous material
AC[c], Amorphous carbon without an identifiable 2D′ peak

Table S5. Peaks in the Raman spectral bands of rocks from the Active Fault Survey Tunnel (sample AFST).

Spot	D [cm^{-1}]	G_{app} [cm^{-1}]	2D [cm^{-1}]	D + D' [cm^{-1}]	2D' [cm^{-1}]	D'_{inf} [cm^{-1}]	D'_{inf} - G_{app} [cm^{-1}]	Species
1	1335.6	1603.2	2708.5	2936.7	3184.3	1592.1	-11.1	GOCM[a]
2	1334.9	1602.9	2648.2	2928.0	3191.9	1595.9	-6.9	GOCM[a]
3	1333.7	1602.8	2655.5	2929.5	3199.7	1599.9	-2.9	GOCM[a]
4	1336.7	1603.3	2679.7	2922.1	3203.7	1601.8	-1.5	GOCM[a]
5	1336.1	1603.7	2658.2	2928.0	3199.1	1599.6	-4.1	GOCM[a]
6	1338.8	1602.5	2447.5	2927.9	3194.7	1597.4	-5.1	GOCM[a]
7	1334.0	1603.2	2595.3	2934.2	3204.8	1602.4	-0.8	GOCM[a]
8	1336.0	1603.3	2675.9	2921.0	3199.6	1599.8	-3.5	GOCM[a]
9	1334.9	1603.3	2667.9	2926.3	3196.6	1598.3	-5.0	GOCM[a]
10	1332.0	1603.0	2689.1	2925.6	3202.3	1601.1	-1.8	GOCM[a]

GOCM[a], graphene oxide–like carbonaceous material

Table S6. Peaks in the Raman spectral bands of rocks from the Atotsugawa area.

Sample	Spot	D [cm^{-1}]	G_{app} [cm^{-1}]	2D [cm^{-1}]	D + D' [cm^{-1}]	2D' [cm^{-1}]	D'_{inf} [cm^{-1}]	D'_{inf} - G_{app} [cm^{-1}]	Species
1124	1	-	-	-	-	-	-	-	Non carbon
	2	-	-	-	-	-	-	-	Non carbon
	3	1320.0	1602.2	-	-	-	-	-	AC[c]
	4	1317.5	1598.9	-	-	-	-	-	AC[c]
	5	-	-	-	-	-	-	-	Non carbon
	6	-	-	-	-	-	-	-	Non carbon
	7	-	-	-	-	-	-	-	Non carbon
	8	1245.0	1603.4	-	-	-	-	-	AC[c]
	9	-	-	-	-	-	-	-	Non carbon
	10	-	-	-	-	-	-	-	Non carbon
1141	1	1331.5	1602.9	2694.5	2936.2	3196.3	1598.1	-4.7	GOCM[a]
	2	1332.9	1604.9	2770.3	2929.1	-	-	-	AC[c]
	3	1332.4	1604.0	2639.6	2938.0	3212.7	1606.3	2.3	RGOCM[b]
	4	1332.9	1603.7	2654.8	2915.4	3208.6	1604.3	0.6	RGOCM[b]
	5	1332.9	1603.8	2645.6	2922.3	3199.9	1600.0	-3.8	GOCM[a]
	6	1329.0	1601.8	-	-	-	-	-	AC[c]
	7	-	-	-	-	-	-		Non carbon
	8	1334.4	1603.8	2591.9	2885.1	3189.5	1594.8	-9.0	GOCM[a]
	9	1330.6	1604.3	2672.3	2921.1	3211.4	1605.7	1.4	RGOCM[b]
	10	1332.7	1604.0	2659.2	2921.0	3200.9	1600.4	-3.6	GOCM[a]

GOCM[a], graphene oxide–like carbonaceous material
RGOCM[b], reduced graphene oxide–like carbonaceous material
AC[c], Amorphous carbon without an identifiable 2D′ peak

Reference

71. Geospatial Information Authority of Japan. GSI website (2024). https://maps.gsi.go.jp/
